# Association between air pollution exposure, physical activity, and risk for cardiometabolic multimorbidity incidence: a cohort study from China

**DOI:** 10.1007/s00484-025-03122-z

**Published:** 2026-01-21

**Authors:** Zihao Wan, Shanshan Cai

**Affiliations:** 1 Faculty for Physical Education, Shanghai International Studies University, Shanghai, China; 2 Division of Biomedical and Life Sciences, Faculty of Health and Medicine, Lancaster University, Lancaster, LA1 4YG UK

**Keywords:** Cardiometabolic multimorbidity, Air pollution, Physical activity, Mediating effect, Elderly health

## Abstract

Cardiometabolic multimorbidity (CMM) has emerged as a major threat to health among older adults, yet the effects of air pollution and physical activity on CMM remain insufficiently investigated. This study aimed to examine the associations between air pollutant exposure, physical activity, and CMM risk, and to analyze the potential moderating and mediating roles of physical activity in the relationship between pollutants and CMM. This study utilized data from the China Health and Retirement Longitudinal Study (CHARLS) from 2015-2020, including 17,718 participants. We assessed exposure levels to PM_2.5_, PM_10_, NO_2_, SO_2_, CO, and O_3_, and categorized physical activity into four levels (Q1-Q4) based on metabolic equivalent quartiles. Cox regression models were employed to analyze the effects of pollutants and physical activity metabolic equivalents on CMM, with restricted cubic splines for dose-response analysis. Additionally, Baron & Kenny's method was applied to evaluate the mediating effect of physical activity. During the follow-up period, 741 participants developed CMM. After multivariate adjustment, all pollutants were significantly associated with increased CMM risk, with SO_2_ (HR=2.768, 95% CI: 2.526-3.033) showing the most significant impact. Higher levels of physical activity demonstrated pronounced protective effects. Dose-response analyses revealed non-linear relationships between pollutants and CMM (P for nonlinear < 0.001), while physical activity exhibited a protective non-linear relationship with CMM (P for nonlinear = 0.037). Mediation analysis identified significant partial mediating effects of physical activity in the relationships between pollutants (PM_2.5_, PM_10_, NO_2_, and O_3_) and CMM, with mediation proportions ranging from 5.71% to 19.88%, with NO_2_ showing the highest mediation proportion (19.88%). Long-term exposure to air pollutants is significantly associated with increased CMM risk, while higher levels of physical activity confer substantial protective effects. Pollutants may partially increase CMM risk indirectly by inhibiting physical activity.

**Supplementary Information:**

The online version contains supplementary material available at 10.1007/s00484-025-03122-z.

## Introduction

Cardiometabolic multimorbidity (CMM), typically defined as the coexistence of at least two cardiometabolic diseases (such as heart disease, stroke, and diabetes) (Han et al. 2021; Valderas et al. 2009), has emerged as a major global public health challenge. With accelerating global population aging, the incidence of CMM shows a continuous upward trend, not only significantly increasing patients’ mortality risk but also imposing a heavy burden on healthcare systems (Adair et al. 2014; Zhang et al. 2019). Research indicates that compared to those with a single disease, CMM patients may have more than twice the risk of death, along with markedly decreased quality of life (Zhang et al. 2019). Previous studies have identified smoking, sedentary behavior, and other behaviors as risk factors for CMM (Chudasama et al. 2020), but many other potential causes remain unexplored.

In recent years, environmental factors, especially air pollution, have been recognized as important risk factors for cardiovascular and metabolic diseases. Numerous studies have confirmed that long-term exposure to air pollutants is associated with increased risk of individual diseases such as coronary heart disease, stroke, and diabetes (Chudasama et al. 2020; Ning et al. 2024). However, research on the relationship between air pollutant exposure and the risk of CMM development is relatively limited, particularly among populations in developing countries. Specifically, the UREP survey observed that for every 10 µg/m³ increase in PM_2.5_ concentration, the risk of CMM increased by 2.2%−7.6%. Similarly, a Chinese cohort study found that each 10 µg/m³ increase in PM_2.5_ concentration was associated with a 17.9% increase in CMM risk (Chudasama et al. 2020). Physical activity, as a modifiable lifestyle factor, plays an important role in preventing and managing cardiometabolic diseases. Many studies have found that sufficient physical activity can reduce inflammation levels, enhance insulin sensitivity, and improve lipid metabolism, thereby reducing the incidence of cardiovascular disease and diabetes (German et al. 2021; Valenzuela et al. 2023). However, the complex relationship among air pollution, physical activity, and CMM risk has not been thoroughly studied, particularly the potential moderating and mediating effects of physical activity in the relationship between air pollution and CMM.

To address these gaps, this study utilizes longitudinal follow-up data from the China Health and Retirement Longitudinal Study (CHARLS) to investigate the associations between air pollutant exposure, physical activity levels, and CMM risk, and to analyze the potential moderating and mediating roles of physical activity in the relationship between pollutants and CMM. Our results will provide important evidence for understanding the comprehensive impact of environmental factors and physical activity on cardiometabolic health in older adults, and provide scientific basis for developing more effective public health intervention strategies.

## Materials and methods

### Research subjects

This study utilizes data from the China Health and Retirement Longitudinal Study (CHARLS) for analysis. CHARLS began in 2011, with its survey coverage spanning 125 cities across China and follow-up visits conducted every 2–3 years. The design concept and methodological framework of this survey have been detailed in relevant literature (Zhao et al. 2014). The research protocol was approved by the Ethics Review Committee of Peking University (IRB00001052-11015), with all respondents providing written informed consent. The research procedures strictly adhered to the ethical guidelines stipulated in the 1964 Declaration of Helsinki.

As the CHARLS database only comprehensively collected physical activity-related data between 2015 and 2020, this study selected tracking data from three time points—2015, 2018, and 2020—for longitudinal analysis (K. Zhang et al. 2025a, b; Zhou et al. 2023). The initial sample included 25,419 participants. We extracted key variables including demographic characteristics (gender, age, etc.), health behaviors (smoking, alcohol consumption status), chronic diseases (heart disease, stroke, diabetes), and physical activity. The study excluded participants diagnosed with cardiovascular diseases or diabetes at baseline (3,131 individuals), those younger than 45 years of age, and participants with missing environmental factors and baseline information, ultimately including 17,718 subjects for analysis (Figure S1).

### Assessment of physical activity

The CHARLS questionnaire categorizes physical activity into vigorous activity (such as lifting weights, digging), moderate-intensity activity (such as cycling, mopping), and light activity/walking (including walking during work, housework, and daily travel). The metabolic equivalent (MET) values for these three types of physical activities are 8.0, 4.0, and 3.3 METs/hour, respectively (Ding et al. 2021; Tian And Shi 2022). Subjects reported the frequency and duration of each activity per week, with duration classified into five levels: 0 min, 10–29 min, 30–119 min, 120–239 min, and ≥ 240 min. We calculated the total metabolic equivalent per week (Total MET-minutes/week) using the formula “MET value × duration (minutes, using the median value of each interval) × weekly frequency. (Ainsworth et al. 2000)” Specifically, the five duration intervals used 0, 20, 75, 180, and 300 min, respectively, as the basis for calculation. Based on quartile analysis, participants were divided into four physical activity level groups: Q1 group (≤ 1732.5 MET-min/week, as the reference group), Q2 group (1732.6–4158 MET-min/week), Q3 group (4158.1–9864 MET-min/week), and Q4 group (> 9864 MET-min/week).

### Definition of CMM

Our study defines Cardiometabolic Multimorbidity (CMM) as having at least two of the three cardiometabolic diseases: heart disease, stroke, and diabetes (Xie et al. 2022; Zhu et al. 2023). Disease status assessment was based on self-reported information from subjects, determined through standardized questionnaires asking, “Has a doctor ever informed you that you have heart disease, stroke, or diabetes/elevated blood sugar (including impaired glucose tolerance and elevated fasting blood glucose)?”

### Environmental exposure assessment

Data for PM_2.5_, PM_10_, NO_2_, SO_2_, CO, and O_3_ were sourced from the China High Air Pollutants (CHAP) dataset (Wei, Li, Lyapustin, et al. 2021; Wei et al. 2023; Wei, Li, Xue, et al. 2021; Yang et al. 2025). This dataset was constructed by integrating dense ground-based observation networks, satellite remote sensing, atmospheric reanalysis, and multi-model simulation data. The study utilized monitoring data from 2015 to 2020, with PM_2.5_, PM_10_, and O_3_ having a high spatial resolution of 1 km × 1 km, while NO_2_, SO_2_, and CO had a spatial resolution of 10 km × 10 km. The coefficient of determination (R^2^) obtained through ten-fold cross-validation for each pollutant indicator ranged between 0.74 and 0.93, indicating good reliability and predictive capability of the data. The study estimated pollutant concentration data at the city level based on participants’ residential addresses and calculated the average exposure levels during the one-year period before the start or end of the study.

### Covariates

This study incorporated multiple covariates based on previous research (Chen et al. 2023; Peng et al. 2024), including demographic characteristics (age, gender), body mass index (BMI), socioeconomic factors (education level, marital status, place of residence, retirement status), geographical location, and lifestyle habits (smoking, alcohol consumption). Additionally, information on sleep duration, mental health (depression score), and history of chronic diseases (hypertension and pulmonary disease) was collected. These variables were primarily used for subsequent sensitivity analyses to verify the robustness of the model results.

### Statistical analysis

We applied repeated measures ANOVA for continuous variables (such as age, pollutant concentrations, physical activity) and chi-square tests for categorical variables (such as gender, smoking, residence) to analyze baseline characteristics.

We employed Cox proportional hazards regression models with time-varying exposures to quantify the relationship between air pollutants (PM_2.5_, PM_10_, NO_2_, SO_2_, CO, and O_3_), physical activity metabolic equivalents, and CMM risk. We established three different models. Model 1 was a crude model without adjustments. Model 2 adjusted for age, gender, and BMI. Model 3 further adjusted for education level, marital status, place of residence, retirement status, geographical location, smoking, and alcohol consumption variables. Furthermore, we conducted stratified analyses by physical activity levels (Q1-Q4), testing the strength of association between environmental exposure and CMM risk in each stratum to verify the moderating effect of physical activity. We assessed the interaction effects between pollutants and physical activity by adding interaction terms to the models.

To evaluate the non-linear relationships between air pollutants (PM_2.5_, PM_10_, NO_2_, SO_2_, CO, and O_3_), physical activity metabolic equivalents, and CMM risk, we employed Restricted Cubic Splines (RCS) for dose-response analysis. The knots in the RCS models were placed at the 10th, 50th, and 90th percentiles of the variable distribution, with the 10th percentile serving as the reference point. Additionally, RCS analyses were conducted for all pollutants across different physical activity levels to assess whether the dose-response relationships between pollutants and CMM changed across different physical activity levels.

We analyzed whether PM_2.5_, PM_10_, NO_2_, and O_3_ affected CMM risk through the mediation of physical activity, applying the Baron & Kenny stepwise method for evaluation (Baron And Kenny 1986; Birhanu et al. 2022). Direct effects reflected the independent impact of pollutants on CMM, while indirect effects demonstrated the pathway through which pollutants influenced CMM via physical activity. The mediation proportion was calculated to determine the strength and relative importance of physical activity in the process of pollutant-induced CMM.

### Sensitivity analyses

We conducted several sensitivity analyses to test the robustness of the results. First, we reassessed the model results by separately adding four covariates, which included hypertension, lung disease, sleep duration, and depression score. Second, we conducted a nested case-control study using the current dataset, where participants with CMM were assigned to the case group, while the control group was selected through propensity score matching with a caliper value of 0.02 and a matching ratio of 1:3. Factors considered during the matching process included age, gender, BMI, education level, marital status, residence, retirement status, geographical location, smoking, hypertension, lung disease, sleep duration, and alcohol consumption. Third, to address potential concerns about the validity of quartile-based classification thresholds, we reassessed the association using the International Physical Activity Questionnaire (IPAQ) classification standards (Chu et al. 2015; Macfarlane et al. 2011; Mou et al. 2025). Participants were categorized into three levels of activity: low level (< 600 MET-minutes/week), moderate level (600–3000 MET-minutes/week), and high level (> 3000 MET-minutes/week). Fourth, to validate the robustness of our mediation analysis findings, we conducted sensitivity analysis using bootstrap methods with 10,000 resampling iterations to calculate bias-corrected confidence intervals for indirect effects, complementing our primary Baron & Kenny approach (Mackinnon et al. 2004).

All statistical analyses were performed using R software (version 4.4.1), with P value < 0.05 considered statistically significant.

## Results

We recruited a total of 17,718 participants with a total follow-up time of 35,198 person-years, among which 741 individuals (4.2%) had CMM (Table 1 and Table S1). The mean age of the CMM group (64.87 ± 9.16 years) was significantly higher than the non-CMM group (61.13 ± 9.66 years) (*P* < 0.001), while BMI showed no significant difference between the two groups (*P* = 0.084). Regarding environmental exposure, except for CO, other pollutants showed significant differences between the two groups (*P* < 0.05). In terms of physical activity, the total metabolic equivalent (MET) in the CMM group (4438.57 ± 5051.17) was significantly lower than in the non-CMM group (6476.22 ± 6257.24) (*P* < 0.001). The proportion of people with the lowest activity level (Q1) was higher in the CMM group (44.5%), while the proportion of people with the highest activity level (Q4) was lower (13.1%) (*P* < 0.001). Table S2 shows the Spearman correlations between all pollutants, indicating strong positive correlations among them. Among these, PM_2.5_, PM_10_, and NO_2_ had correlation coefficients greater than 0.8, showing stronger correlations.

**Table 1:** Baseline characteristics of participants

Characteristic	Overall	CMM		*p*-value
		No	Yes	
Population, *n*				
No. of participants	17,718	16,977	741	
**Demographic factors**,** mean (SD)**				
Age (years)	61.28(9.67)	61.13(9.66)	64.87(9.16)	< 0.001
BMI (kg/m^2^)	22.14(2.98)	22.13(2.97)	22.33(3.06)	0.084
**Environmental factors**,** mean (SD)**				
PM_2.5_	36.67(13.52)	36.57(13.50)	38.76(13.92)	< 0.001
PM_10_	64.90(30.90)	64.64(30.53)	70.72(37.99)	< 0.001
NO_2_	24.44(8.34)	24.39(8.31)	25.51(8.97)	< 0.001
SO_2_	13.86(7.26)	13.89(7.32)	13.19(5.75)	0.01
CO	0.84(0.20)	0.84(0.20)	0.83(0.19)	0.178
O_3_	96.09(11.48)	95.95(11.45)	99.51(11.74)	< 0.001
**Physical Activity**				
Total MET, mean (SD)	6391.00(6224.76)	6476.22(6257.24)	4438.57(5051.17)	< 0.001
**Physical Activity Group**,** n (%)**				
Q1 (≤ 1732.5)	5484(31.0)	5154(30.4)	330(44.5)	< 0.001
Q2 (1732.6–4158)	3622(20.4)	3457(20.4)	165(22.3)	
Q3 (4158.1–9864)	4182(23.6)	4033(23.8)	149(20.1)	
Q4 (> 9864)	4430(25.0)	4333(25.5)	97(13.1)	

Abbreviations: CMM, cardiometabolic multimorbidity; PM_2.5_, atmospheric particulate matter with a kinetic diameter less than or equal to 2.5 micrometers; PM_10_, atmospheric particulate matter with a kinetic diameter less than or equal to 10 micrometers; NO_2_, nitrogen dioxide; SO_2_, sulphur dioxide; CO, carbon monoxide; O_3_, ozone

Age, BMI and environmental factors are presented as mean (standard) deviation; other variables are presented as numbers (percentages)

Table 2 demonstrates the association between air pollutants and CMM risk. In three progressively adjusted Cox models, all studied pollutants were significantly associated with CMM risk (*P* < 0.001). In model 3, PM_2.5_ (HR = 1.444, 95% CI: 1.386–1.504), NO_2_ (HR = 1.620, 95% CI: 1.517–1.731), and SO_2_ (HR = 2.768, 95% CI: 2.526–3.033) showed strong associations with CMM risk, while PM_10_ (HR = 1.103, 95% CI: 1.092–1.115) and O_3_ (HR = 1.107, 95% CI: 1.055–1.161) showed relatively weaker associations. Each 1 µg/m^3^ increase in CO (HR = 1.712, 95% CI: 1.600–1.831.600.831) was associated with a 71.2% increase in risk. Overall, long-term exposure to air pollutants was significantly associated with CMM risk, with SO_2_ showing the highest risk.

**Table 2:** Associations between PM_2.5_, PM_10_, NO_2_, SO_2_, CO, O_3_ and CMM

Exposure	Model 1		Model 2		Model 3	
	HR (95% CI)	*p*-value	HR (95% CI)	*p*-value	HR (95% CI)	*p*-value
PM_2.5_	1.415 (1.359, 1.474)	< 0.001	1.434 (1.377, 1.494)	< 0.001	1.444 (1.386, 1.504)	< 0.001
PM_10_	1.107 (1.095, 1.118)	< 0.001	1.110 (1.098, 1.122)	< 0.001	1.103 (1.092, 1.115)	< 0.001
NO_2_	1.607 (1.506, 1.716)	< 0.001	1.639 (1.535, 1.751)	< 0.001	1.620 (1.517, 1.731)	< 0.001
SO_2_	2.619 (2.391, 2.868)	< 0.001	2.714 (2.478, 2.971)	< 0.001	2.768 (2.526, 3.033)	< 0.001
CO	1.649 (1.544, 1.763)	< 0.001	1.693 (1.583, 1.810)	< 0.001	1.712 (1.600, 1.831)	< 0.001
O_3_	1.087 (1.037, 1.141)	< 0.001	1.106 (1.055, 1.160)	< 0.001	1.107 (1.055, 1.161)	< 0.001
Model 1, crude model;						
Model 2, adjusted for age, gender, BMI;						
Model 3, adjusted for age, gender, BMl, marital status, Retirement status, residence, education level, region, smoking and drinking.						

Abbreviations: CMM, cardiometabolic multimorbidity; PM_2.5_, atmospheric particulate matter with a kinetic diameter less than or equal to 2.5 micrometers; PM_10_, atmospheric particulate matter with a kinetic diameter less than or equal to 10 micrometers; NO_2_, nitrogen dioxide; SO_2_, sulphur dioxide; CO, carbon monoxide; O_3_, ozone; HR, hazard ratio; CI, confidence interval

Table 3 shows the association between physical activity levels and CMM. In model 3, compared to Q1, all higher levels of physical activity were significantly associated with reduced CMM risk. Q2 physical activity level was associated with a 17.4% reduction in CMM risk (HR = 0.826, 95% CI: 0.722–0.945, *P* = 0.005), Q3 level was associated with a 33.5% reduction (HR = 0.665, 95% CI: 0.577–0.766, *P* < 0.001), while the highest activity level Q4 was associated with a 49.3% reduction (HR = 0.507, 95% CI: 0.429–0.600.429.600, *P* < 0.001). Trend tests showed a significant downward trend in CMM risk with increasing levels of physical activity (P for trend < 0.001). This suggests that higher levels of physical activity may have a protective effect against CMM.

**Table 3:** Association between physical activity levels and CMM. Physical activity was categorized as Q1 (≤ 1732.5 MET-min/week, reference), Q2 (1732.6–4158 MET-min/week), Q3 (4158.1–9864 MET-min/week) and Q4 (> 9864 MET-min/week)

Physical activity	Model 1		Model 2		Model 3	
	HR (95% CI)	*p*-value	HR (95% CI)	*p*-value	HR (95% CI)	*p*-value
Level						
Q1	1	Ref	1	Ref	1	Ref
Q2	0.805 (0.705, 0.919)	0.001	0.852 (0.745, 0.974)	0.019	0.826 (0.722, 0.945)	0.005
Q3	0.589 (0.512, 0.678)	< 0.001	0.647 (0.562, 0.745)	< 0.001	0.665 (0.577, 0.766)	< 0.001
Q4	0.393 (0.334, 0.463)	< 0.001	0.462 (0.391, 0.545)	< 0.001	0.507 (0.429, 0.600)	< 0.001
*P* for trend		< 0.001		< 0.001		< 0.001

**Fig. 1:**
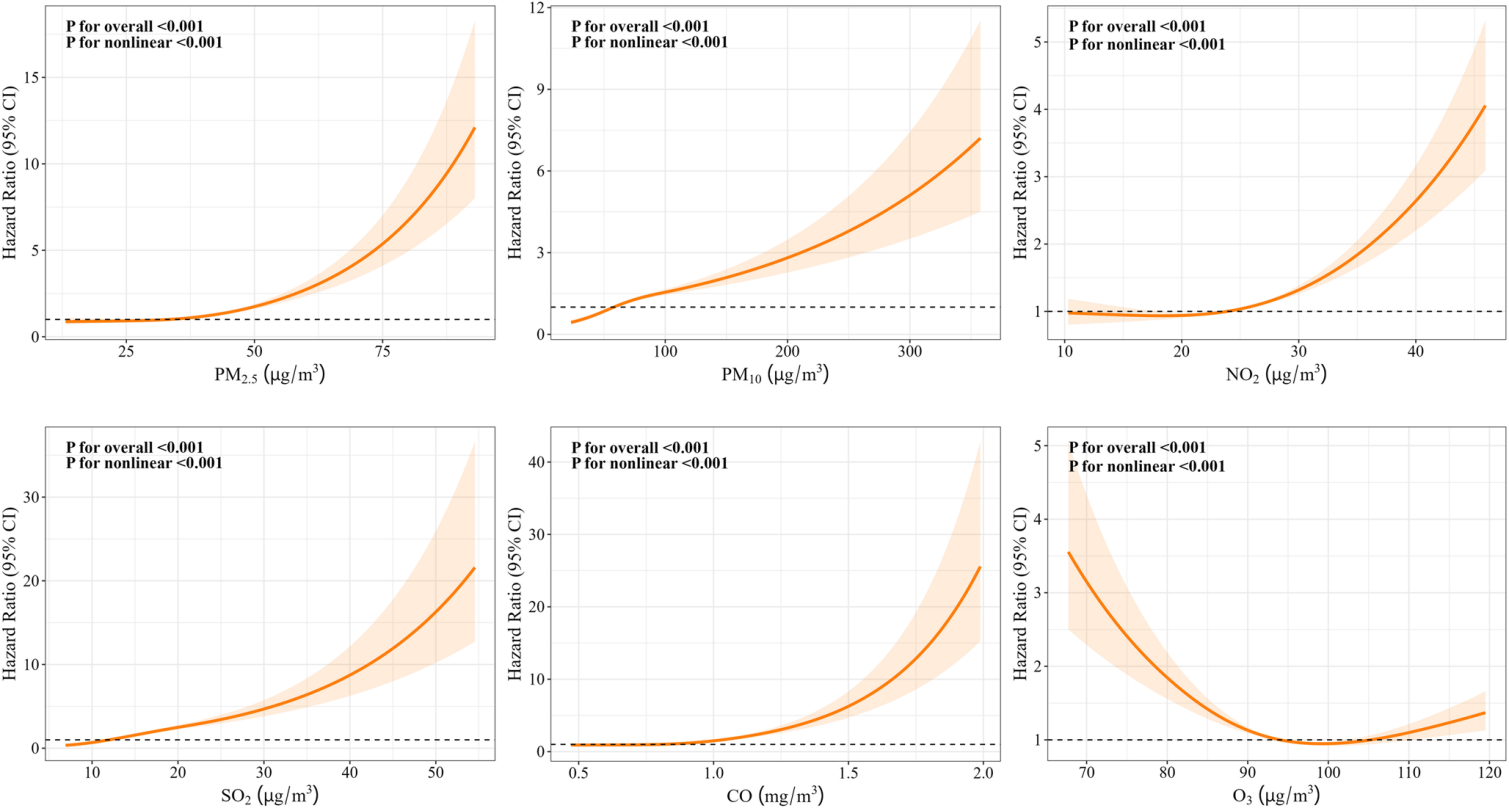
Dose-response relationships between PM_2.5_, PM_10_, NO_2_, SO_2_, CO, O_3_ and CMM. Models were adjusted for age, gender, BMI, marital status, retirement status, residence, education level, place of residence, smoking status, and alcohol consumption

**Fig. 2:**
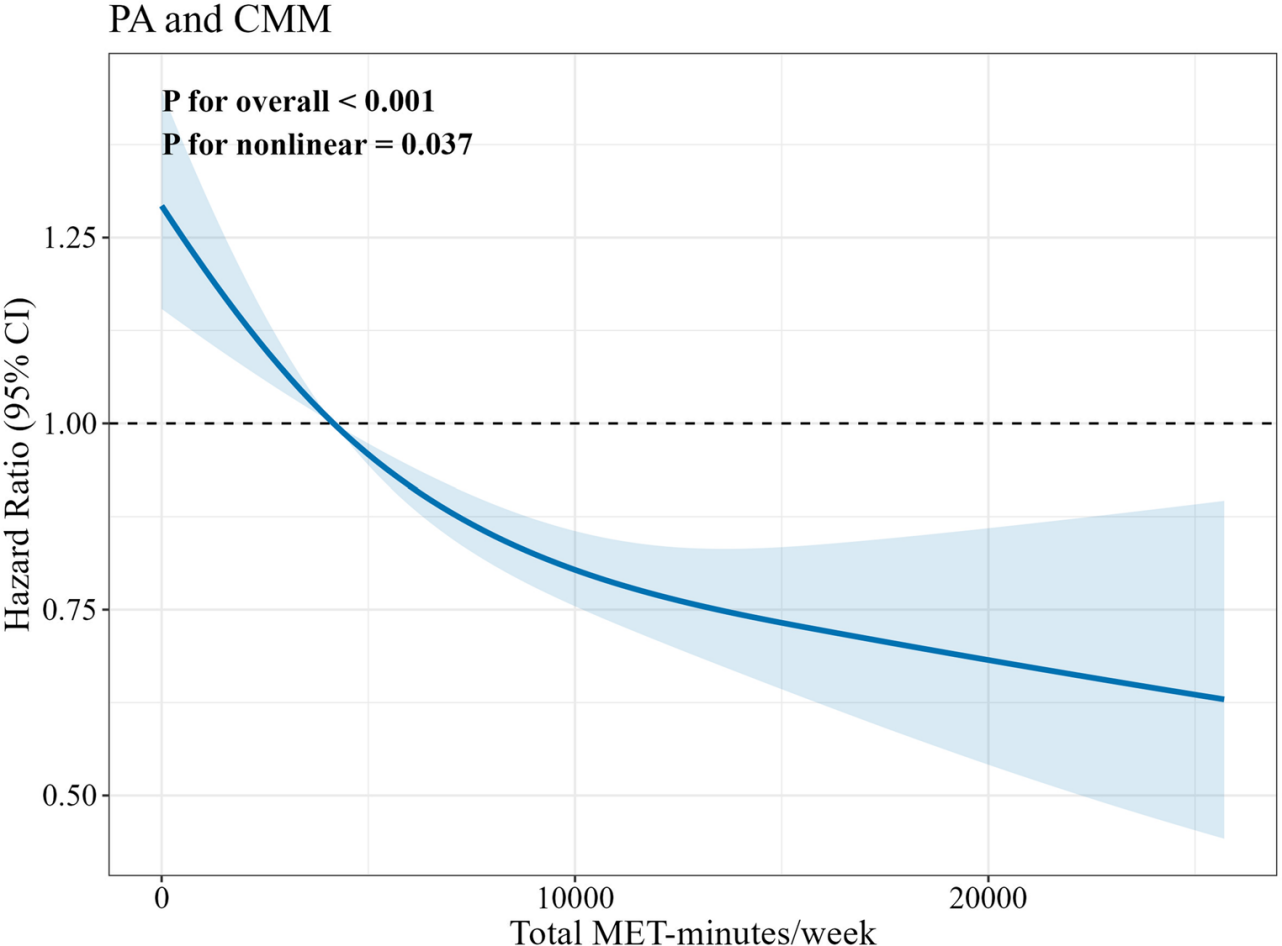
Dose-response relationships between physical activity and CMM. Models were adjusted for age, gender, BMI, marital status, retirement status, residence, education level, place of residence, smoking status, and alcohol consumption

Figure 1 illustrates the non-linear dose-response relationship between air pollutants and CMM. All studied pollutants showed statistically significant overall associations with CMM risk (P for overall < 0.001), and these associations exhibited non-linear characteristics (P for nonlinear < 0.001). Figure 2 reveals a significant protective dose-response relationship between physical activity and CMM, with CMM risk showing a non-linear downward trend as weekly total metabolic equivalents increased (P for overall < 0.001, P for nonlinear = 0.037), indicating that physical activity has a protective effect on cardiometabolic health. In the interaction analysis between physical activity levels and pollutant exposure (Figure S2-7), we found that in all physical activity level groups (Q1-Q4), increases in all pollutant concentrations except O_3_ were associated with increased CMM risk (P for overall < 0.05). PM_10_, SO_2_, and CO showed non-linear characteristics in Q2-Q4 groups (P for nonlinear < 0.05).

**Fig. 3:**
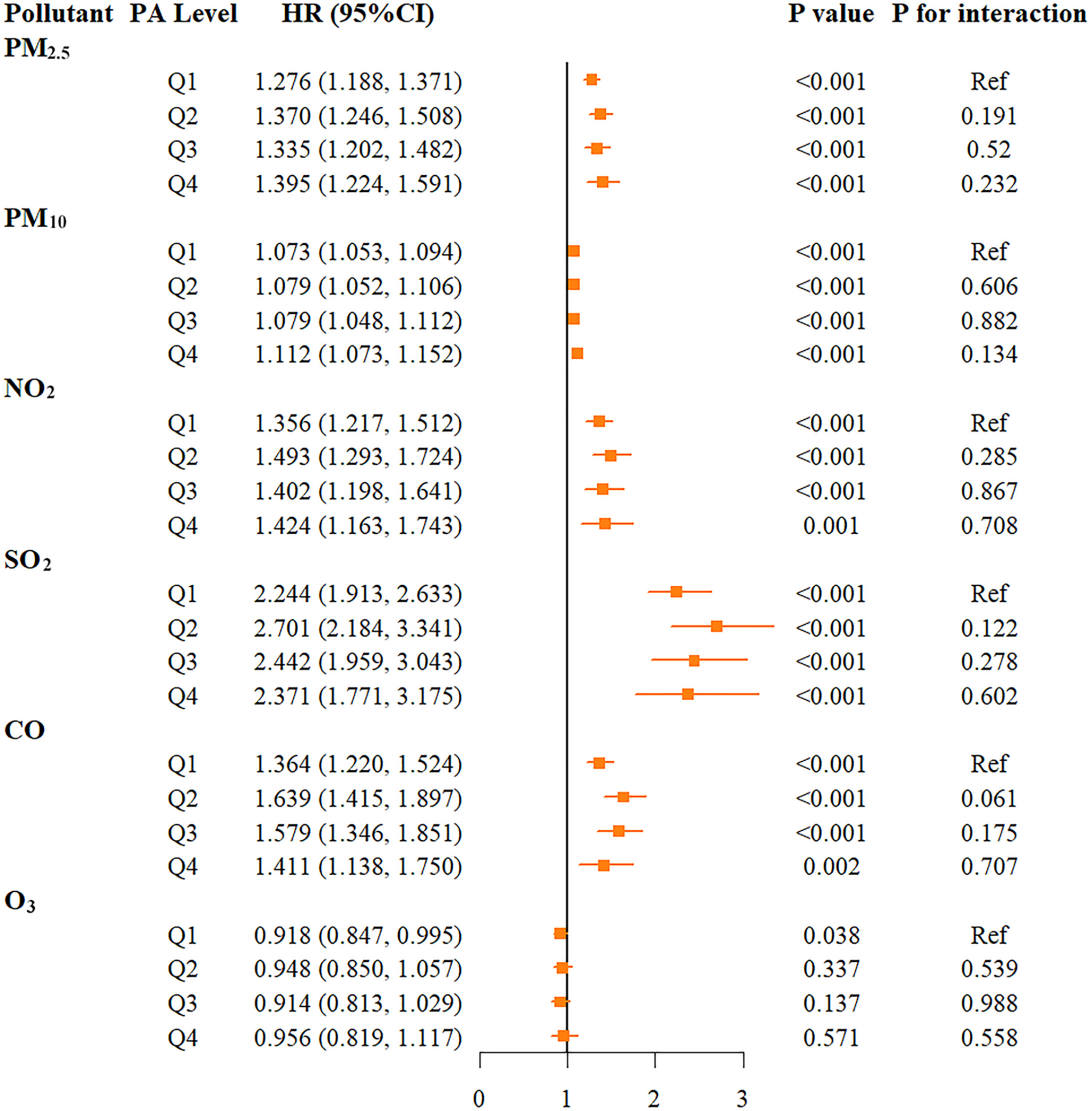
Risk for diabetes incidence associated with PM_2.5_, PM_10_, NO_2_, SO_2_, CO, O_3_ stratified by physical activity levels. Models were adjusted for age, gender, BMI, marital status, retirement status, residence, education level, place of residence, smoking status, and alcohol consumption

Figure 3 shows the association between air pollutants and CMM risk at different physical activity levels (*P* < 0.001). For PM_2.5_ and PM_10_, CMM risk increased with higher physical activity levels. For PM_2.5_, the risk in the Q4 group (HR: 1.395, 95% CI: 1.224–1.591) was higher than in the Q1 group (HR: 1.276, 95% CI: 1.188–1.371). For SO_2_, NO_2_, and CO, CMM risk was highest in the Q2 group. The impact of SO_2_ on CMM risk was most significant, with the HR in the Q2 group at 2.701 (95% CI: 2.184–3.341), higher than other groups. The HRs for NO_2_ and CO were 1.493 (95% CI: 1.293–1.724) and 1.639 (95% CI: 1.415–1.897), respectively. The HR for O_3_ in the Q1 group was 0.918 (95% CI: 0.847–0.995, *P* = 0.038), while it did not reach statistical significance in other activity level groups. However, interaction analysis indicated that the interactions between pollutants and physical activity levels did not reach statistical significance (P interaction > 0.05).

**Fig. 4:**
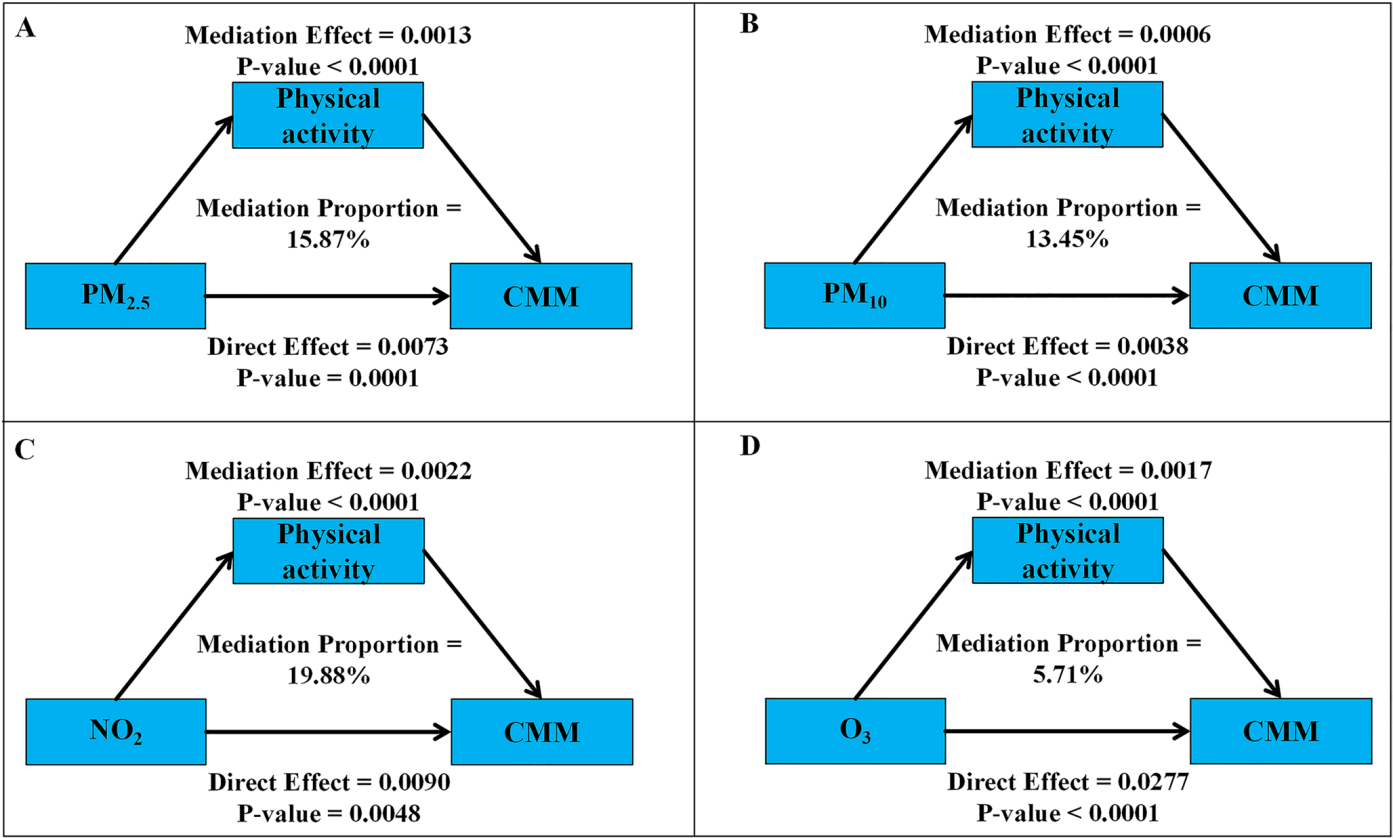
Mediation analysis of physical activity in the association between PM_2.5_, PM_10_, NO_2_, O_3_ and CMM

We conducted mediation analysis on the relationships between PM_2.5_, PM_10_, NO_2_, O_3_ and CMM risk, with physical activity as a mediator (Fig. 4). We observed that PM_2.5_, PM_10_, NO_2_, and O_3_ had significant mediating effects on CMM incidence through physical activity (*P* < 0.001), with mediation proportions ranging from 5.71% to 19.88%. NO_2_ had the highest mediation proportion (19.88%), while O_3_ had the lowest (5.71%). The mediation proportions for PM_2.5_ and PM_10_ were 15.87% and 13.45%, respectively. All pollutants showed significant direct effects on CMM (*P* < 0.001), with O_3_ having the highest direct effect (0.0277). These results suggest that pollutants may partially increase CMM risk by inhibiting physical activity.

Sensitivity analysis verified the robustness of our study. After adding other covariates in model 3, all pollutants still showed significant associations with CMM (Table S3). We conducted a nested case-control study, including 1,423 new CMM cases and matching 4,234 participants without CMM as controls at a ratio of 1:3. We found that except for O_3_, the results for other pollutants were generally consistent with the main analysis results (Table S4). When using IPAQ classification standards, the results were generally consistent with the main quartile-based analysis, with moderate level (HR: 0.760, 95% CI: 0.654–0.884) and high level (HR: 0.617, 95% CI: 0.537–0.709) physical activity both showing significant protective effects against CMM compared to low level activity (Table S5). Bootstrap sensitivity analysis with 10,000 iterations yielded results consistent with the Baron & Kenny approach, confirming significant mediation effects for all pollutants with indirect effects ranging from 0.0181 to 0.0216 and mediation proportions ranging from 8.72% to 13.67% (Figure S8).

## Discussion

Based on CHARLS data, we explored the complex relationship between air pollutant exposure, physical activity levels, and CMM risk. Our results indicate that long-term exposure to air pollutants increases CMM risk, while higher levels of physical activity can reduce CMM risk. Additionally, we found that physical activity has a partial mediating effect in the relationship between pollutants and CMM.

Previous studies have confirmed an association between pollutants and increased risk of CMM (Cui et al. 2024; Jiang et al. 2023; Peng et al. 2024; Zou et al. 2023). A prospective cohort study from China found that for every 10 µg/m³ increase in PM_2.5_, CMM risk increased by 17.9% (Peng et al. 2024). A UK Biobank study found that for each IQR increase in PM_2.5_, PM_10_, and NO_2_, the risk of CMM mortality increased by 11% (−2% to 26%), 22% (7% to 38%), and 33% (17% to 51%) (Jiang et al. 2023), respectively. Two other studies also found similar associations (Cui et al. 2024; Zou et al. 2023). Furthermore, our research provides strong evidence for the protective effect of physical activity against CMM. Two UK Biobank studies showed that moderate to high-intensity physical activity could reduce the risk of coexistence of diabetes and cardiovascular disease (Liu et al. 2023; Wang et al. 2025).

Regarding the potential mechanisms by which CMM is affected by pollutants and physical activity, relatively few studies currently exist. Most perspectives suggest that particulate matter can cross the alveolar-blood barrier into the bloodstream, triggering systemic oxidative stress responses, disrupting cardiac function, and accelerating atherosclerosis (Fiordelisi et al. 2017; Fouladi et al. 2020; Haberzettl et al. 2016). Gaseous pollutants can activate the autonomic nervous system, increase sympathetic nervous activity, causing vasoconstriction and reduced heart rate variability (Beckett et al. 1985; Felber Dietrich et al. 2008). Pollutants may influence gene expression through epigenetic mechanisms. PM_2.5_ exposure can alter DNA methylation patterns, leading to long-term metabolic disorders (Lei et al. 2019). The protective effect of physical activity on CMM may involve multiple interrelated molecular and cellular mechanisms. Moderate physical activity can significantly improve endothelial function, enhance the body’s antioxidant capacity, and reduce oxidative stress damage (Salem et al. 2025; Zhang et al. 2025a, b). Additionally, in terms of metabolic regulation, physical activity can promote mitochondrial biogenesis and improved function, enhance insulin sensitivity, promote energy metabolism, and regulate lipid transport (Mølmen et al. 2025; Sepehri et al. 2025). However, the exact mechanisms merit clarification in future research.

Regarding whether physical activity in polluted air environments remains beneficial, controversy still exists (D’Oliveira et al. 2023; Tainio et al. 2021). A Chinese study found that long-term exercise could partially mitigate the negative impact of pollutant exposure on ischemic heart disease (Raza et al. 2021). A UK Biobank study found that physical activity provided benefits for diabetes patients at different pollution levels, but like our research results, did not observe an interaction between pollutants and physical activity (Li et al. 2022). Similar to our findings, a SALSA study found that in populations with higher levels of outdoor physical activity, each 10 ppb increase in O_3_ concentration increased diabetes risk by 52% (Yu et al. 2021). Notably, O_3_ demonstrated a unique pattern where overall analysis indicated increased CMM risk, yet stratified analysis revealed protective effects only in the lowest physical activity group, suggesting effect modification by activity levels. This may reflect O_3_’s dual role whereby potential hormetic benefits occur at low exposure during minimal exertion, while enhanced respiratory uptake during increased activity overwhelms protective mechanisms (Devlin et al. 2012; Juneja Gandhi et al. 2022; Yu et al. 2021). These differences may be related to factors such as study population characteristics, pollutant composition, and physical activity measurement methods. In our study, we found that in the high physical activity level group, the risk effects of some pollutants (such as PM_2.5_ and PM_10_) on CMM were slightly higher than in the low activity level group, suggesting that when engaging in vigorous activity in highly polluted environments, the body’s intake of pollutants may increase, potentially offsetting some of the benefits of physical activity. This phenomenon can be explained by the physiological changes during exercise: physical activity increases minute ventilation by 10–20 fold compared to resting state, with concurrent increases in respiratory rate and tidal volume, leading to enhanced deposition of inhaled pollutants in both upper and lower respiratory tracts (Carlisle And Sharp 2001; Daigle et al. 2003). Additionally, during exercise, individuals tend to shift from nasal to oral breathing, bypassing the natural filtration mechanisms of the nasal cavity and allowing greater penetration of fine particles into the lungs (Rundell And Caviston 2008; Rundell et al. 2008). Furthermore, exercise-induced bronchodilation and increased alveolar ventilation may enhance the absorption and systemic distribution of gaseous pollutants and ultrafine particles (Miller et al. 2017; Tainio et al. 2021). An intriguing paradox emerged in our findings regarding SO_2_ exposure. The CMM group exhibited lower mean SO_2_ levels, yet SO_2_ showed the highest hazard ratio among all pollutants. Several explanations may account for this discrepancy. First, a “healthy survivor effect” may exist whereby high-risk individuals in heavily polluted areas have relocated or already progressed to severe disease stages (Pearce et al. 2007). Second, CMM patients may intentionally reduce outdoor activities during high-pollution periods, lowering measured exposure (An et al. 2018). Third, SO_2_ may co-occur with unmeasured pollutants in industrial regions (Thurston et al. 2017). Fourth, SO_2_ may exhibit steeper dose-response relationships at lower concentrations compared to particulate matter (Orellano et al. 2021). Fifth, city-level exposure assessment may introduce misclassification due to SO_2_’s heterogeneous spatial distribution (Peng et al. 2006; Wu et al. 2020). Finally, residual confounding from socioeconomic factors and healthcare access cannot be excluded. These findings underscore the need for future studies with individual-level exposure assessment and comprehensive co-pollutant measurements. Through mediation analysis, we discovered for the first time that physical activity has a partial mediating effect in the relationship between pollutants and CMM, with mediation proportions ranging from 5.71% to 19.88%. This finding has received little attention in previous research. Pollutants may affect physical activity through multiple pathways. On one hand, polluted weather may lead to recommendations to reduce outdoor activities, increasing sedentary behavior (An et al. 2019; Zhan et al. 2023). On the other hand, long-term exposure to pollutants may damage respiratory system function, leading to decreased exercise endurance, thereby limiting physical activity capacity (Madureira et al. 2019).

This study has several significant advantages. First, it is based on a large-scale nationally representative cohort data, including 17,718 middle-aged and elderly people from 125 cities across China, with a large sample size and strong representativeness. Second, it included multiple air pollutant indicators, comprehensively assessed the impact of environmental exposure, and explored the moderating role of physical activity. Fourth, it employed mediation analysis to investigate potential mechanisms by which pollutants affect CMM.

This study also has some limitations. First, the assessment of disease and physical activity was based on self-reporting, which may involve recall bias and underestimate the actual impact. Additionally, CMM status lacked clinical or biochemical validation, potentially missing undiagnosed cases. Second, due to data limitations, the study could not distinguish between indoor and outdoor physical activity, which may have significant differences in terms of pollution exposure. Additionally, air pollution exposures were not assessed at the individual level but estimated using city-level data based on residential addresses, which could lead to non-differential misclassification of exposure. However, this should typically bias pollutant-related health impacts towards the null directions. Third, this exposure assessment approach also did not account for indoor pollution sources, individual mobility patterns, and occupational exposures. Fourth, several potential confounding factors were not adjusted for, including dietary factors and healthcare accessibility, which may influence both physical activity and CMM risk.

## Conclusion

We found that long-term exposure to air pollutants is significantly associated with increased risk of CMM, while higher levels of physical activity have a significant protective effect. Furthermore, physical activity has a partial mediating effect in the relationship between pollutants and CMM, suggesting that pollutants may indirectly affect health by inhibiting physical activity. These findings emphasize the dual importance of improving environmental quality and promoting appropriate physical activity in preventing cardiometabolic multimorbidity.

Future research should focus on mechanistic studies to elucidate biological pathways linking air pollutants, physical activity, and cardiometabolic health, as well as intervention trials examining whether structured exercise programs can mitigate pollution-related health risks. From a public health perspective, integrated policies simultaneously addressing environmental quality and physical activity promotion are needed. Future guidelines should consider pollution levels when recommending outdoor activities, and personalized interventions accounting for individual exposure and activity capacity may optimize disease prevention strategies.

## Supplementary Information

Below is the link to the electronic supplementary material.

Supplementary Material 1 (DOCX 6.96 MB)

## Data Availability

The datasets supporting the conclusions of this article is available in the website of China Health and Retirement Longitudinal Study (https://charls.pku.edu.cn/).
