# Supplementary material for: Association between air pollution exposure, physical activity, and risk for cardiometabolic multimorbidity incidence: a cohort study from China
Source: Int J Biometeorol. 2026 Jan 21;70(2):34. doi: 10.1007/s00484-025-03122-z (PMC12823677; doi:10.1007/s00484-025-03122-z)
Supplement: Supplementary file 1 — Supplementary Material 1 (DOCX 6.96 MB) [file 484_2025_3122_MOESM1_ESM.docx]

Supplementary Material

# **1.**Supplementary Tables

**Table S1** Baseline characteristics of participants.

| **Characteristic** | | | **Overall** | **CMM** | | ***p*-value** |
| --- | --- | --- | --- | --- | --- | --- |
|  |  |  |  | **No** | **Yes** |  |
| **Population** | | |  |  |  |  |
| No. of participants | | | 17718 | 16977 | 741 |  |
| Cumulative observations | | | 35198 | 33774 | 1424 |  |
| **Demographic factors** | | |  |  |  |  |
| Age (years) | | | 62.00(9.58) | 61.78(9.64) | 64.42(8.54) | <0.001 |
|  | | <65 | 11278(63.7) | 10932(64.4) | 346(46.7) | <0.001 |
|  | | ≥65 | 6440(36.3) | 6045(35.6) | 395(53.3) |  |
| BMI (kg/m^2^) | | | 24.86(30.23) | 24.78(31.45) | 25.80(8.96) | 0.173 |
|  | | Underweight | 1669(9.4) | 1610(9.5) | 59(8.0) | 0.200 |
|  | | Normalweight | 13111(74.0) | 12565(74.0) | 546(73.7) |  |
|  | | Overweight | 2938(16.6) | 2802(16.5) | 136(18.4) |  |
| Gender | | |  |  |  | 0.03 |
|  | Female | | 9077(51.2) | 8668(51.1) | 409(55.2) |  |
|  | Male | | 8641(48.8) | 8309(48.9) | 332(44.8) |  |
| Marital status | | |  |  |  | <0.001 |
|  | | other | 2444(13.8) | 2303(13.6) | 141(19.0) |  |
|  | | Married | 15274(86.2) | 14674(86.4) | 600(81.0) |  |
| Retirement status | | |  |  |  |  |
|  | | Non-retired | 15026(84.8) | 14469(85.2) | 557(75.2) |  |
|  | | Retired | 2692(15.2) | 2508(14.8) | 184(24.8) |  |
| Residence | | |  |  |  | <0.001 |
|  | | Rual | 7204(40.7) | 6842(40.3) | 362(48.9) |  |
|  | | Urban | 10514(59.3) | 10135(59.7) | 379(51.1) |  |
| Region (South) | | |  |  |  | <0.001 |
|  | | South | 10148(57.3) | 9852(58.0) | 296(39.9) |  |
|  | | North | 7570(42.7) | 7125(42.0) | 445(60.1) |  |
| **Health status** | | |  |  |  |  |
| Smoking | | |  |  |  | <0.001 |
|  | | No | 12707(71.7) | 12124(71.4) | 583(78.7) |  |
|  | | Yes | 5011(28.3) | 4853(28.6) | 158(21.3) |  |
| Drinking (No) | | |  |  |  | <0.001 |
|  | | No | 11154(63.0) | 10608(62.5) | 546(73.7) |  |
|  | | Yes | 6564(37.0) | 6369(37.5) | 195(26.3) |  |

Abbreviations: CMM, cardiometabolic multimorbidity; PM_2.5_, atmospheric particulate matter with a kinetic diameter less than or equal to 2.5 micrometers; PM_10_, atmospheric particulate matter with a kinetic diameter less than or equal to 10 micrometers; NO_2_, nitrogen dioxide; SO_2_, sulphur dioxide; CO, carbon monoxide; O_3_, ozone.

Age, BMI and environmental factors are presented as mean ± standard deviation; other variables are presented as numbers (percentages).

**Table S2** Spearman correlations between annual mean PM_2.5_, PM_10_, NO_2_, SO_2_, CO, and O_3_ concentrations during the follow-up period.

| **Exposure** | PM_2.5_ | PM_10_ | NO_2_ | SO_2_ | CO | O_3_ |
| --- | --- | --- | --- | --- | --- | --- |
| PM_2.5_ | 1 | 0.957 | 0.848 | 0.611 | 0.728 | 0.417 |
| PM_10_ |  | 1 | 0.848 | 0.683 | 0.705 | 0.483 |
| NO_2_ |  |  | 1 | 0.548 | 0.68 | 0.526 |
| SO_2_ |  |  |  | 1 | 0.733 | 0.035 |
| CO |  |  |  |  | 1 | 0.137 |
| O_3_ |  |  |  |  |  | 1 |

Abbreviations: PM_2.5_, atmospheric particulate matter with a kinetic diameter less than or equal to 2.5 micrometers; PM_10_, atmospheric particulate matter with a kinetic diameter less than or equal to 10 micrometers; NO_2_, nitrogen dioxide; SO_2_, sulphur dioxide; CO, carbon monoxide; O_3_, ozone.

**Table S3** Associations between PM_2.5_, PM_10_, NO_2_, SO_2_, CO, O_3_ and CMM after adjusting extra covariates.

| Exposure | HR (95% CI) | | | | | | | | |
| --- | --- | --- | --- | --- | --- | --- | --- | --- | --- |
|  | Main model |  | + Hypertension |  | + Lung Disease |  | + Sleep duration |  | + CESD10 |
| PM_2.5_ | 1.444 (1.386, 1.504) |  | 1.404 (1.347, 1.463) |  | 1.454 (1.396, 1.514) |  | 1.454 (1.396, 1.515) |  | 1.463 (1.405, 1.524) |
| PM_10_ | 1.103 (1.092, 1.115) |  | 1.096 (1.084, 1.107) |  | 1.098 (1.087, 1.110) |  | 1.103 (1.092, 1.115) |  | 1.101 (1.090, 1.112) |
| NO_2_ | 1.620 (1.517, 1.731) |  | 1.554 (1.456, 1.660) |  | 1.660 (1.554, 1.773) |  | 1.657 (1.551, 1.770) |  | 1.700 (1.590, 1.817) |
| SO_2_ | 2.768 (2.526, 3.033) |  | 2.707 (2.470, 2.965) |  | 2.810 (2.564, 3.080) |  | 2.832 (2.584, 3.103) |  | 2.820 (2.574, 3.090) |
| CO | 1.712 (1.600, 1.831) |  | 1.691 (1.580, 1.809) |  | 1.742 (1.628, 1.863) |  | 1.738 (1.624, 1.859) |  | 1.751 (1.637, 1.874) |
| O_3_ | 1.107 (1.055, 1.161) |  | 1.068 (1.018, 1.120) |  | 1.127 (1.074, 1.183) |  | 1.121 (1.068, 1.176) |  | 1.130 (1.077, 1.185) |

**Table S4** Associations between PM_2.5_, PM_10_, NO_2_, SO_2_, CO, O_3_ and CMM based on a nested case-control study.

| Exposure | HR (95% CI) | | |
| --- | --- | --- | --- |
|  | Main model |  | nested case-control study |
| PM_2.5_ | 1.444 (1.386, 1.504) |  | 1.355 (1.252, 1.467) |
| PM_10_ | 1.103 (1.092, 1.115) |  | 1.145 (1.097, 1.196) |
| NO_2_ | 1.620 (1.517, 1.731) |  | 1.474 (1.311, 1.658) |
| SO_2_ | 2.768 (2.526, 3.033) |  | 3.262 (2.488, 4.277) |
| CO | 1.712 (1.600, 1.831) |  | 1.464 (1.301, 1.647) |
| O_3_ | 1.107 (1.055, 1.161) |  | 0.944 (0.864, 1.032) |

**Table S5** Association between physical activity levels and CMM. Physical activity was categorized according to IPAQ classification standards as Low (< 600 MET-min/week, reference), Moderate (600-3000 MET-min/week), and High (> 3000 MET-min/week).

| Physical activity | Model 1 | | Model 2 | | Model 3 | |
| --- | --- | --- | --- | --- | --- | --- |
|  | HR (95% CI) | *p*-value | HR (95% CI) | *p*-value | HR (95% CI) | *p*-value |
| Level |  |  |  |  |  |  |
| Low | 1 | Ref | 1 | Ref | 1 | Ref |
| Moderate | 0.775 (0.668, 0.898) | 0.001 | 0.827 (0.713, 0.959) | 0.012 | 0.760 (0.654, 0.884) | <0.001 |
| High | 0.533 (0.465, 0.610) | <0.001 | 0.615 (0.535, 0.706) | <0.001 | 0.617 (0.537, 0.709) | <0.001 |
| *P* for trend |  | <0.001 |  | <0.001 |  | <0.001 |

**2.Supplementary Figures**


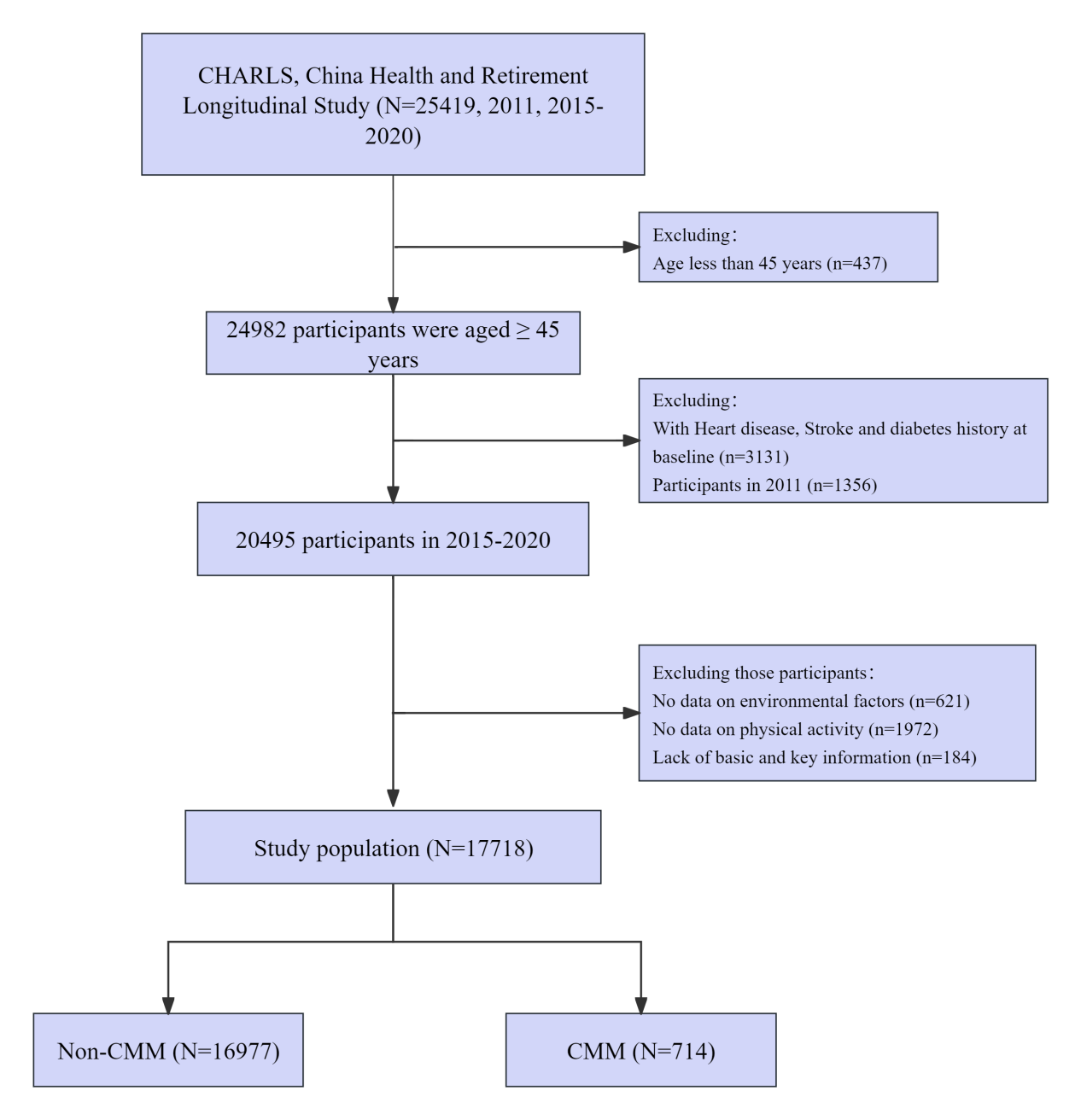


**Figure S1** Flowchart for this study.

**

**

Figure S2 Dose-response relationships between PM_2.5_ and CMM in different physical activity groups.

**

**

Figure S3 Dose-response relationships between PM_10_ and CMM in different physical activity groups.

**

**

Figure S4 Dose-response relationships between NO_2_ and CMM in different physical activity groups.

**

**

Figure S5 Dose-response relationships between SO_2_ and CMM in different physical activity groups.

**

**

Figure S6 Dose-response relationships between CO and CMM in different physical activity groups.

**

**

Figure S7 Dose-response relationships between O_3_ and CMM in different physical activity groups.





Figure S8 Bootstrap mediation analysis of physical activity in the association between PM_2.5_, PM_10_, NO_2_, O_3_ and CMM.
